# Abnormal Cannabidiol Affects Production of Pro-Inflammatory Mediators and Astrocyte Wound Closure in Primary Astrocytic-Microglial Cocultures

**DOI:** 10.3390/molecules25030496

**Published:** 2020-01-23

**Authors:** Julian Cardinal von Widdern, Tim Hohmann, Faramarz Dehghani

**Affiliations:** Department of Anatomy and Cell Biology, Martin Luther University Halle-Wittenberg, 06097 Halle (Saale), Germany; julian.cardinal-von-widdern@medizin.uni-halle.de (J.C.v.W.); tim.hohmann@medizin.uni-halle.de (T.H.)

**Keywords:** abnormal cannabidiol, astrocytes, cannabinoid ligands, inflammation, interleukin-6, microglia, neuroinflammation, nitric oxide, synthetic cannabinoids, tumor necrosis factor α

## Abstract

Abnormal cannabidiol (abn-CBD) exerts neuroprotective effects in vivo and in vitro. In the present study, we investigated the impact of abn-CBD on the glial production of proinflammatory mediators and scar formation within in vitro models. Primary astrocytic-microglial cocultures and astrocytic cultures from neonatal C57BL/6 mice and CB_2_ receptor knockout mice were stimulated with lipopolysaccharide (LPS), and the concentrations of tumor necrosis factor α (TNFα), interleukin-6 (IL-6) and nitrite were determined. Furthermore, we performed a live cell microscopy-based scratch-wound assay. After LPS stimulation, TNFα, IL-6 and nitrite production was more strongly increased in cocultures than in isolated astrocytes. Abn-CBD treatment attenuated the LPS-induced production of TNFα and nitrite in cocultures, while IL-6 production remained unaltered. In isolated astrocytes, only LPS-induced TNFα production was reduced by abn-CBD. Similar effects were observed after abn-CBD application in cocultures of CB_2_ knockout mice. Interestingly, LPS-induced TNFα and nitrite levels were far lower in CB_2_ knockout cultures compared to wildtypes, while IL-6 levels did not differ. In the scratch-wound assay, treatment with abn-CBD decelerated wound closure when microglial cells were present. Our data shows a differential role of abn-CBD for modulation of glial inflammation and astrocytic scar formation. These findings provide new explanations for mechanisms behind the neuroprotective potential of abn-CBD.

## 1. Introduction

Acute lesions of the central nervous system (CNS), like traumatic brain injury and stroke, are major public health problems [1,2,3,4]. Current treatment options are partially not sufficient, and are restricted to certain time windows [3,5]. Acute CNS lesions share common pathophysiological features. The initial detrimental event causing the primary irreversible neuronal insult is followed by the secondary neuronal damage, which is characterized by complex interlocking inflammatory and metabolic cascades, and can strongly aggravate the loss of neurons. Mechanisms of secondary neuronal damage are excitotoxicity, neuroinflammation, blood brain barrier (BBB) disruption, gliosis and leukocyte invasion [6,7,8,9]. Initially, the inflammatory response is mainly determined by the local activation of microglial cells and astrocytes [10,11,12]. 

Microglial cells accumulate at the lesion site and play a key role in secondary lesion and the initiation of reparative mechanisms. During activation they undergo distinct morphological changes, proliferate, release cytokines and gain the ability of phagocytosis [12,13]. Microglial and astrocytic production of tumor necrosis factor α (TNFα), interleukin 6 (IL-6) and nitric oxide (NO) have been implicated in increased inflammatory status and secondary damage [14,15,16,17,18,19]. Reactive astrogliosis is regularly accompanied by morphological changes and a significantly increased expression of the intermediate glial fibrillary acidic protein (GFAP) [20,21]. However, reactive astrogliosis is a special form of wound healing or scar formation, with the goal of metabolically isolating the damaged region from healthy tissue, reconstructing the BBB, and reorganizing neuronal connections around the lesion [10].

Endocannabinoids (eCBs), such as 2-arachidonyl glycerol (2-AG) and arachidonyl ethanol- amide (AEA) represent a group of lipid mediators that are synthesized on demand and activate G_i/0_-protein-coupled receptors [22]. Predominantly, the effects of eCBs are mediated via cannabinoid receptors such as CB_1_ and CB_2_, which are well characterized at the cellular and molecular level [23]. While CB_1_ receptors are most abundant on neurons, CB_2_ receptors are mainly expressed on immune cells [23,24,25]. In recent years, understanding of the complexity of cannabinoid signaling has increased. Several other cannabinoid-responsive targets as well as eCB-synthesizing and -degrading enzymes have been revealed. Furthermore, several complementary signaling cascades and a biased signaling of classical cannabinoid receptors have been reported [26,27]. The eCB system is widely expressed in the organism, including leukocytes, where it modulates immune function and inflammation [28,29,30].

Cannabinoid receptors, as well as eCBs, are up-regulated during brain lesions, and are related to neuroprotective effects [31,32,33]. There is growing evidence for the eCB system as part of an endogenous compensatory mechanism to reduce secondary lesion growth and promote reparative processes. According to established concepts the neuroprotective effects of eCBs are mediated due to a reduction of synaptic transmission and excitotoxicity via neuronal CB_1_ receptors and decreasing neuroinflammation via CB_2_ receptors on microglia and leukocytes [32,34,35,36,37,38].

Experiments with CB_1_ and CB_2_ double knockout mice suggest the existence of additional non- CB_1_, non-CB_2_ G-protein-coupled cannabinoid receptors. Abnormal cannabidiol (abn-CBD; IUPAC: 4-[(1R,6R)-3-methyl-6-prop-1-en-2-yl-1-cyclohex-2-enyl]-5-pentylbenzene-1,3-diol), a synthetic regioisomer of the phytocannabinoid cannabidiol with very low affinity to the classical cannabinoid receptors, mediates its action via a non-CB_1_, non-CB_2_ target. This receptor has first been described on blood vessels [39,40,41,42,43] and characterized pharmacologically in the CNS on microglial cells promoting cell migration [44,45]. Various orphan G-protein coupled receptors have been discussed as the putative abn-CBD-sensitive receptor. In this context, the receptors GPR18 and GPR55 have been proposed as potential new cannabinoid receptors [46,47,48,49]. There are several lines of evidence that GPR18 plays an important role in the regulation of microglial function [49]. However, the literature must be rated as inconsistent in this regard [50,51]. The complex ligand dependent signal transduction pathways of GRP18 have been proposed as an explanation for the discrepant literature [52].

Previously, in excitotoxically-lesioned organotypic hippocampal slice cultures (OHSCs), we demonstrated the mediation of the neuroprotective effects of 2-AG by abn-CBD-sensitive receptor [53]. 2-AG improved neuronal survival and affected the migration and proliferation of microglial cells. Surprisingly, the 2-AG effects were not counteracted by preincubation with CB_1_ or CB_2_ receptor antagonists, but were reduced by O-1918, an abn-CBD-sensitive receptor antagonist. Besides 2-AG abn-CBD caused neuroprotection and reduction in microglial accumulation at the injury site. These effects were abolished after depletion of microglial cells [53,54]. Abn-CBD exerted neuroprotective effects after focal cerebral ischemia in rats as well [55]. In this model of stroke, the cerebral blood flow was increased by abn-CBD, therefore, the vasoactive properties of the substance were discussed as the cause of neuroprotection. Broadening these findings, our results from the OHSC model imply a direct causal influence on local CNS cells. 

Since there is few data available on abn-CBD effects in astrocytes, we investigated the abn-CBD effects on a) glial production of pro-inflammatory mediators, and b) astrocytic scar formation with special regard to its temporal course in vitro.

## 2. Results

### 2.1. LPS Stimulates the Production of NO, TNFα and IL-6 in Astrocytic-Microglial Cocultures and Isolated Astrocytes

**Figure 1:**
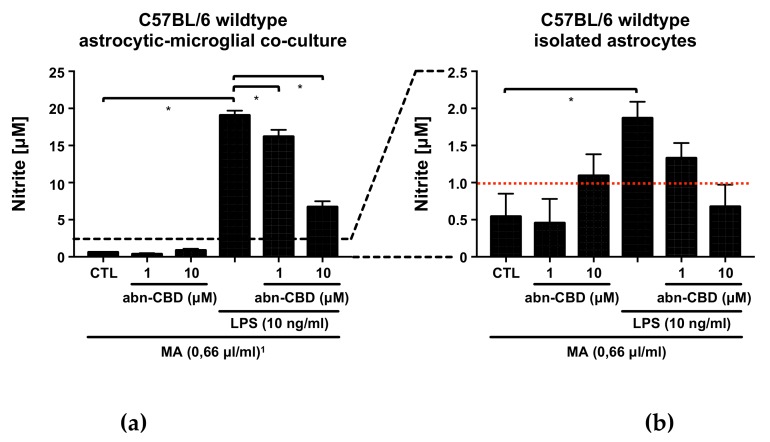

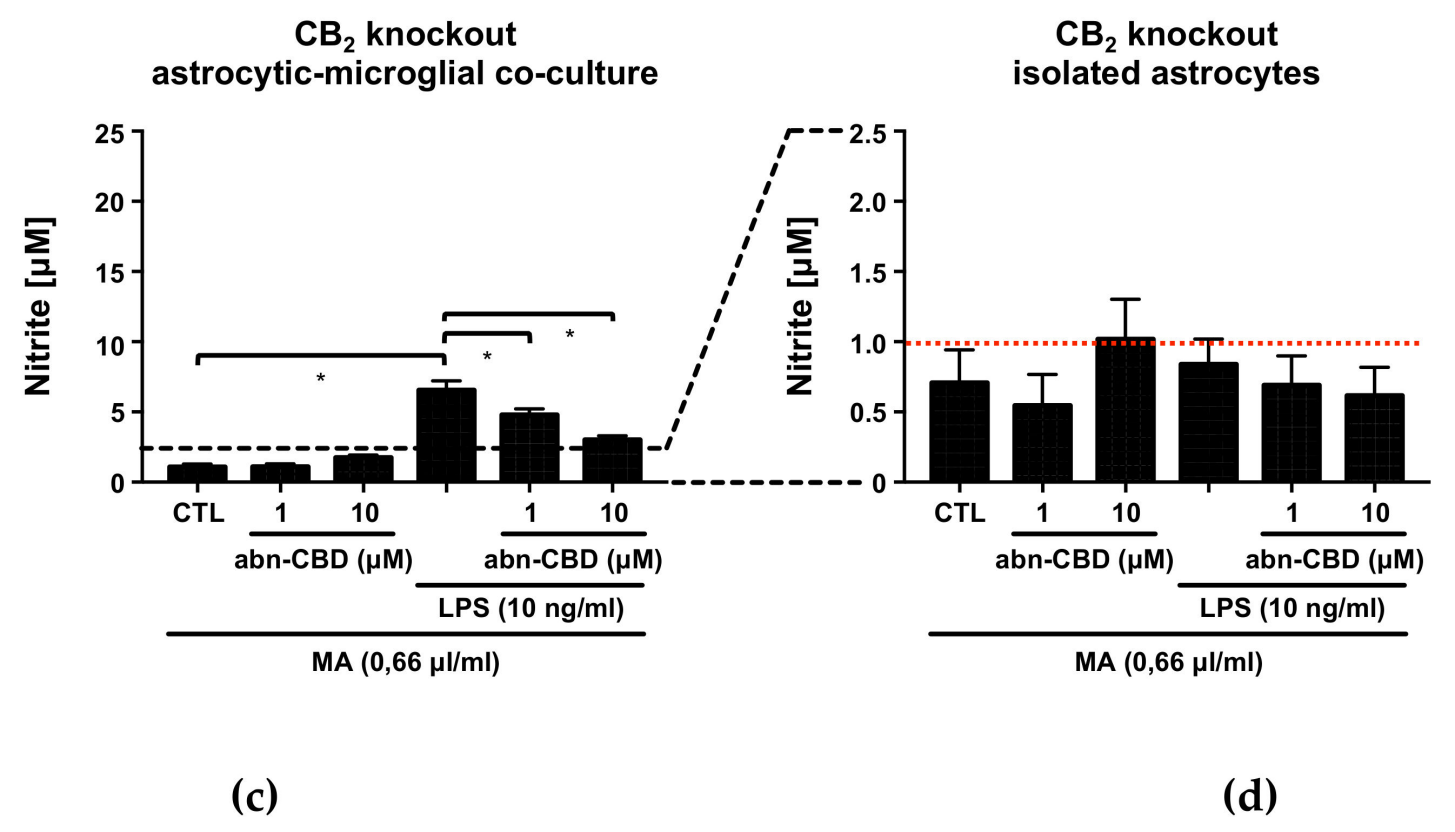
Nitrite measurement in supernatants from astrocytic-microglial cocultures (**a**,**c**) and isolated astrocytes (**b**,**d**) from C57BL/6 wildtype (**a**,**b**) and CB_2_ knockout mice (**c**,**d**). Data is expressed as mean ± standard error of the mean (SEM), n = 12 in each group. Statistical analysis was done using one-way analysis of variance (ANOVA) followed by Bonferroni’s post-test. * p < 0.05. Note the different scaling of the *y*-axis between cocultures (**a**,**c**) and isolated astrocytes (**b**,**d**). The lower detection limit of the assay, as given by the manufacturer and estimated on basis of the standard curve, is shown by the red dotted line. ^1^ All groups received the same concentration (0.66 µL/mL) of the solvent methylacetate (MA) as contained in 10 µM abn-CBD groups.

**Figure 2:**
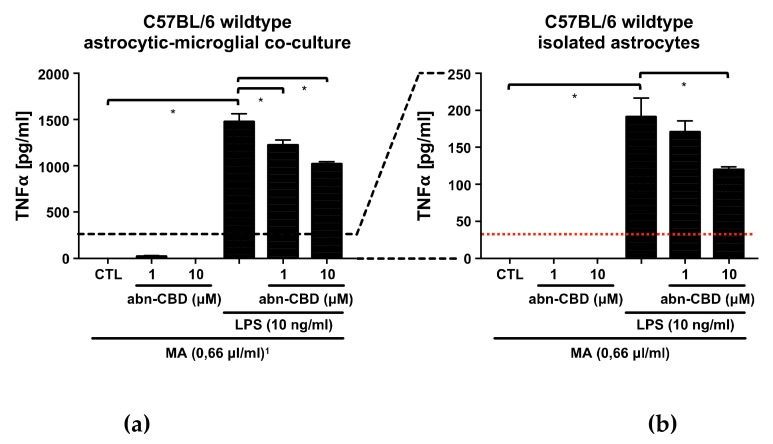

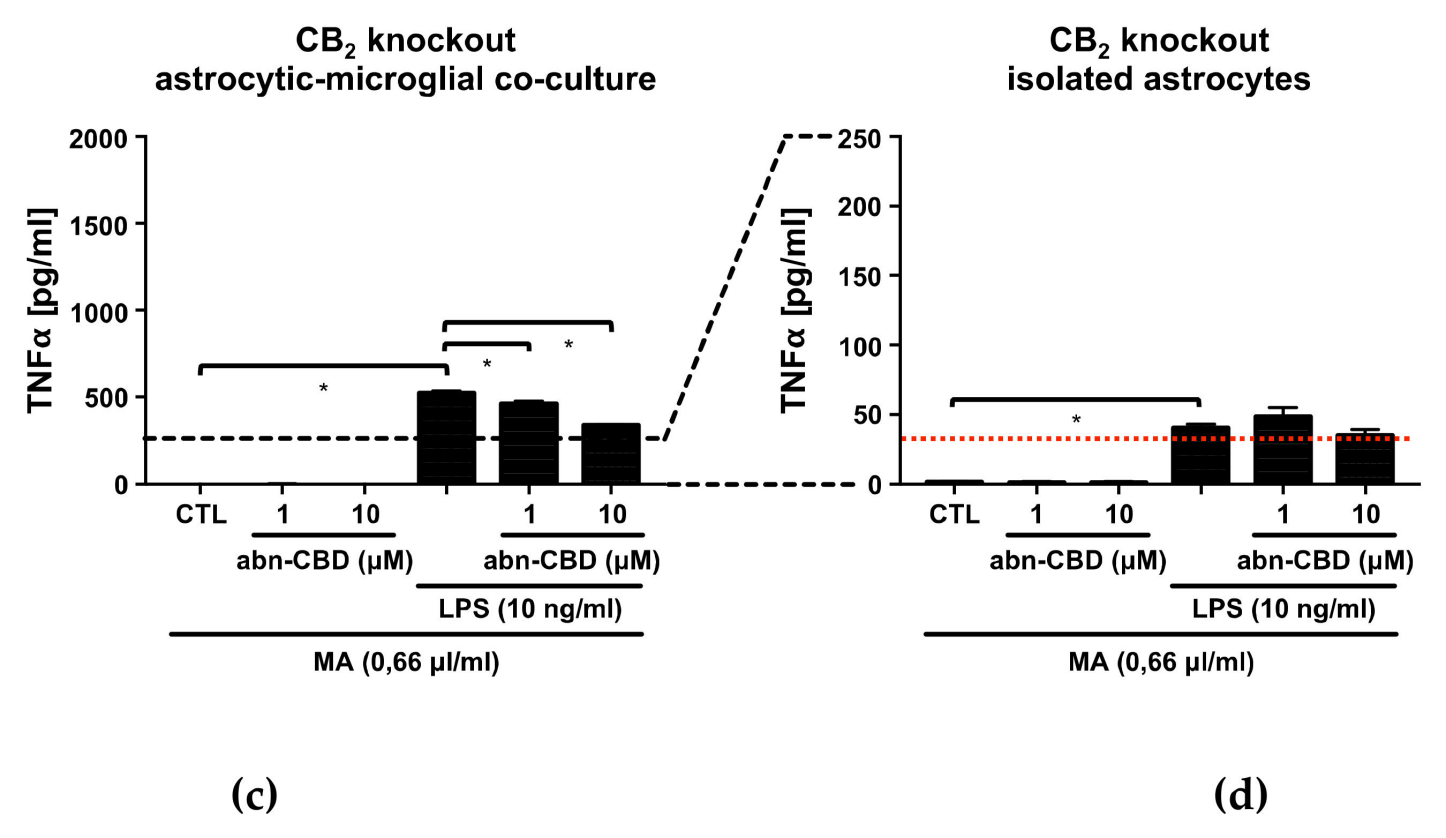
TNFα measurement in supernatants from astrocytic-microglial cocultures (**a**,**c**) and isolated astrocytes (**b**,**d**) from C57BL/6 wildtype (**a**,**b**) and CB_2_ knockout mice (**c**,**d**). Data is expressed as mean ± SEM, n = 8 in each group. Statistical analysis was done using one-way ANOVA followed by Bonferroni’s post-test. * p < 0.05. Note the differing scaling of the *y*-axis between cocultures (**a**,**c**) and isolated astrocytes (**b**,**d**). The lower detection limit of the assay as given by the manufacturer and estimated on basis of the standard curve is shown by the red dotted line. ^1^ All groups received the same concentration (0.66 µL/mL) of the solvent methylacetate (MA) as contained in 10 µM abn-CBD groups.

**Figure 3:**
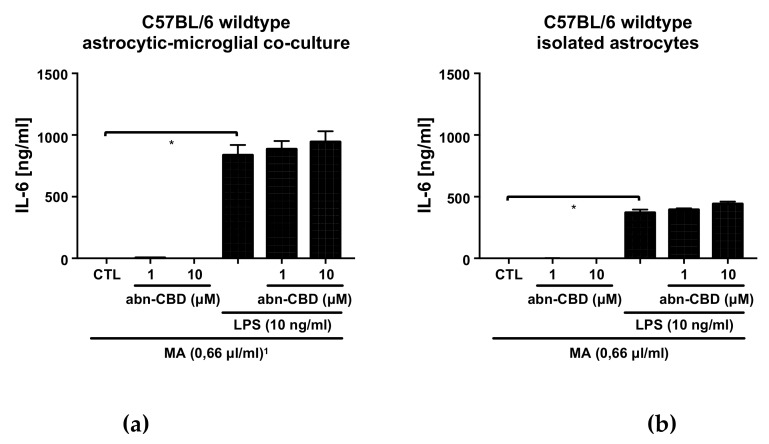

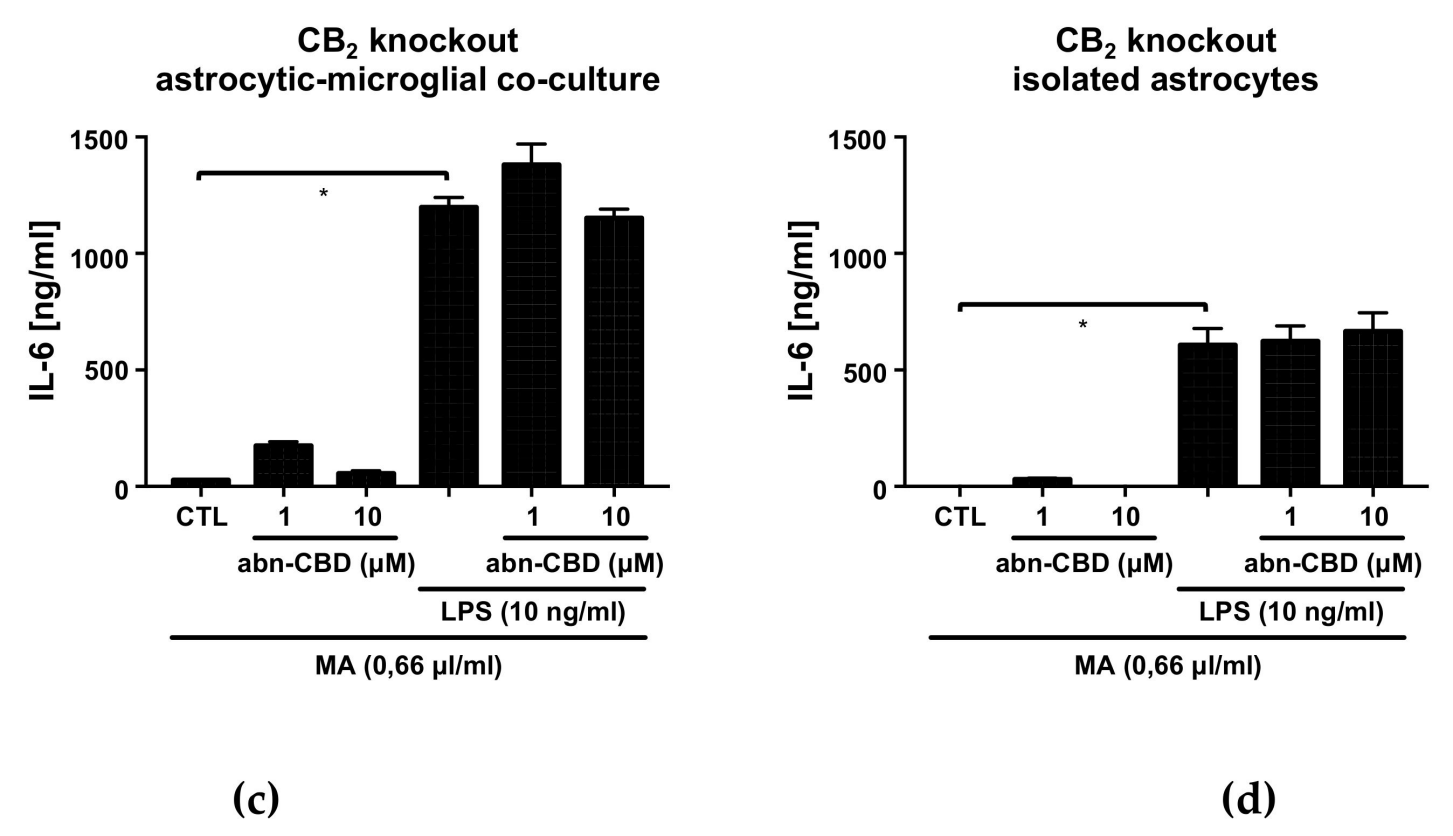
IL-6 measurement in supernatants from astrocytic-microglial cocultures (**a**, **c**) and isolated astrocytes (**b**,**d**) from C57BL/6 wildtype (**a**,**b**) and CB_2_ knockout mice (**c**,**d**). Data is expressed as mean ± SEM, n = 8 in (**a**,**b**) and 4 in (**c**,**d**). Statistical analysis was done using one-way ANOVA followed by Bonferroni’s post-test. * p < 0.05. ^1^ All groups received the same concentration (0.66 µL/mL) of the solvent methylacetate (MA) as contained in 10 µM abn-CBD groups.

In the control groups of all examined cultures, only very low to non-measurable concentrations of nitrite, TNFα and IL-6 were detectable. Treatment with abn-CBD alone had no effect on the baseline production of NO, TNFα or IL-6 (Figure 1, Figure 2 and Figure 3). LPS-treatment led to a strong increase in the production of NO, TNFα and IL-6 in astrocytic-microglial cocultures (Panels a, c, Figure 1, Figure 2 and Figure 3). The isolated wildtype astrocytic cultures were also significantly stimulated, but at much lower levels compared to cocultures (Panels b, d, Figure 1, Figure 2 and Figure 3). However, differences between wildtype cocultures and isolated astrocytic cultures were smaller for IL-6 than for NO and TNFα. Thus, the IL-6 values of the lipopolysaccharide (LPS)-stimulated cocultures differed about the factor of two compared to the astrocytic cultures in contrast to factor 10 in the TNFα and NO values.

### 2.2. Abn-CBD Reduces LPS-Induced Production of NO in Astrocytic-Microglial Cocultures

Since NO in aqueous solution has a half-life of only a few seconds before it reacts further to nitrite and nitrate, the Griess reaction was chosen as a suitable analysis tool to detect nitrite as a stable reaction product [56,57,58]. In LPS-stimulated wildtype cocultures, treatment with abn-CBD resulted in a concentration-dependent reduction of nitrite formation (Figure 1a). Isolated astrocytic cultures from wildtype animals showed no significant reduction of LPS-induced nitrite formation (Figure 1b). Notably, stimulation of astrocytes alone resulted in a very small amount of nitrite at the lower edge of the measurement range. Interestingly, the cultures from CB_2_ knockout animals showed a significantly reduced response to LPS compared to the wildtype (Figure 1c). LPS-induced nitrite formation was reduced by abn-CBD in CB_2_ knockout cocultures in a concentration-dependent manner (Figure 1c). In isolated astrocytes from CB_2_ knockout mice, no significant stimulation was achieved by LPS (Figure 1d).

### 2.3. Abn-CBD Reduces LPS-Induced Production of TNFα in Astrocytic-Microglial Cocultures and Isolated Astrocytic Cultures

In LPS-stimulated wildtype cocultures, treatment with abn-CBD resulted in a concentration-dependent reduction of TNFα production (Figure 2a). In isolated astrocytic cultures, LPS-induced TNFα production was also reduced significantly at the concentration of 10 μM (Figure 2b). The cultures from CB_2_ knockout animals showed a weaker response to LPS compared to the wildtype. In isolated astrocytic cultures from CB_2_ knockout animals, LPS-induced TNFα production was not reduced by abn-CBD, although it should be noted that all values were at the lower end of the measurement range (Figure 2d). Interestingly, the obtained results on TNFα strongly resembled those of NO measurements.

### 2.4. Abn-CBD has No Effect on LPS-Induced IL-6 Production in Astrocytic-Microglial Cocultures and Isolated Astrocytic Cultures

In both LPS-stimulated wildtype cultures, treatment with abn-CBD had no effect on IL-6 production (Figure 3). Cultures from CB_2_ knockout animals showed no altered response to LPS compared to the wildtype. Furthermore, abn-CBD did not affect LPS-stimulated IL-6 production in CB_2_ deficient cultures. Overall, measurements of IL-6 secretion thus showed a different picture compared to the results obtained from TNFα and NO measurement.

### 2.5. Abn-CBD Delays Astrocyte Wound Closure in a Microglia-Dependent Manner

**Figure 4:**
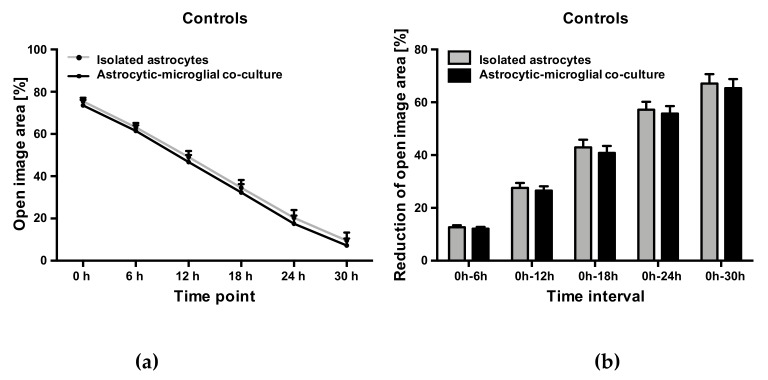
Open image area (**a**) and cumulative reduction of open image area (**b**) in controls from astrocytic-microglial cocultures and isolated astrocytes. Data is expressed as mean ± SEM, n = 8 in each group. Statistical analysis was done using one-way ANOVA followed by Bonferroni’s post-test.

Comparing the determined values of the controls of astrocytic-microglial cocultures and isolated astrocytic cultures, an almost identical wound closure occurred over the observation period. Accordingly, changes in the cell-free area versus the initial wound area did not differ at any time between controls from the two cultures (Figure 4). The presence of microglia in the cultures consequently had no influence on the astrocyte wound closure.

**Figure 5:**
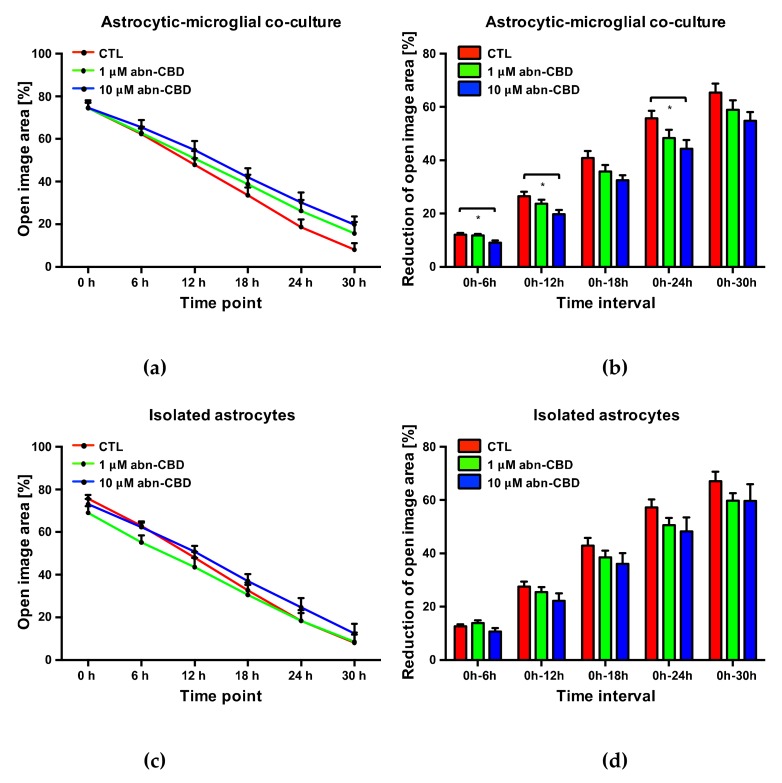
Open image area (**a**,**c**) and cumulative reduction of open image area (**b**,**d**) in astrocytic-microglial cocultures (**a**,**b**) and isolated astrocytes (**c**,**d**). Data is expressed as mean ± SEM, n = 8 in each group. Statistical analysis was done using one-way ANOVA followed by Bonferroni’s post-test. * p < 0.05.

However, the change of free image area compared to the initial wound area was significantly reduced after 6, 12 and 24 h in astrocytic-microglial cocultures treated with 10 μM abn-CBD (Figure 5b, supplementary Video S2). The reduction was not significant after treatment with 1 μM abn-CBD. In isolated astrocytic cultures we observed lower reduction after 10 µM abn-CBD without reaching significant levels (Figure 5d). Abn-CBD affected the astrocytic wound closure most effectively when microglial cells were present in the culture.

**Figure 6:**
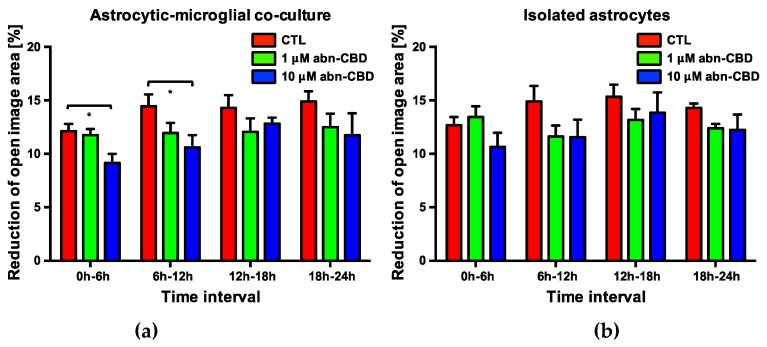
Reduction of open image area in astrocytic-microglial cocultures (**a**) and isolated astrocytes (**b**). Data is expressed as mean ± SEM, n = 8 in each group. Statistical analysis was done using one-way ANOVA followed by Bonferroni’s post-test. * p < 0.05.

In order to further characterize the temporal dynamics of the observed delay in wound closure, an analysis of the change in the cell-free image area at each observed 6-h interval was performed. In cocultures treated with 10 μM abn-CBD, a significant reduction of wound closure was observed in the first two 6-h intervals Figure 6a). There was no significant difference in the subsequent intervals. 1 μM abn-CBD did not trigger this effect. The detected delay in wound closure by 10 μM abn-CBD in astrocytic-microglial cocultures therefore results from an effect within the first twelve hours after injury.

## 3. Discussion

Acute CNS lesions, such as traumatic brain injury (TBI) or stroke are a common cause of persistent neurological failures, cognitive deficits and disability [1,2]. The secondary lesion is partly due to the mechanisms of sterile neuroinflammation, excitotoxicity and oxidative stress [6,7,8,9]. eCBs have been associated with preventing effects on secondary lesion [31,32,33,59]. The eCB system is involved in intrinsic regulation of the local response to a neuronal lesion [31,32,33]. Cannabinoids develop their neuroprotective potential partly through anti-inflammatory effects on astrocytes and microglial cells [60,61,62,63]. Treatment with abn-CBD led to neuroprotective effects in vivo and in vitro, although there is uncertainty about the cellular and molecular mechanisms [53,55,64]. Accordingly, the aim of this study was to further elucidate cellular mechanisms behind abn-CBD-mediated neuroprotection.

In the present in vitro models, the involvement of non-CNS located immune cells is excluded. Hemodynamic or cerebral blood flow influencing events suggested as the cause of the neuroprotective effects of abn-CBD are also excluded [55]. Exemplary immunohistochemistry was carried out to ensure the stability of the composition of the cultures and effectiveness of microglia depletion. No IB_4_-positive microglial cell was detected in isolated astrocytic cultures. In astrocytic-microglial cocultures, the microglial cells were partly clustered and partly localized between the astrocytes. Morphological signs for cell damage such as nuclear condensation or fragmentation were absent in all treatment groups of both cultures. In astrocytic-microglial cocultures IB_4_-positive microglial cells were found in each treatment group (supplementary Figure S1). 

Both astrocytes and microglia express toll-like receptor 4 (TLR4) and are converted to an activated state by LPS [65,66,67]. The activation of TLR4 by LPS, including the intracellular cascades, is well characterized [68]. Glial activation in the context of secondary lesion is also partly mediated by TLR4. After CNS lesion, high-mobility group box protein 1 (HMGB1) and heat shock protein 60 (HSP60) released from dying neurons activate TLR4 on microglia analogous to LPS [69,70,71]. TLR4 activation leads to the production of pro-inflammatory mediators such as NO, TNFα or IL-6. All three substances have in common that they are associated with an inflammatory activated phenotype of microglia, which can be neurotoxic in the context of secondary damage [14,15,16,17,18,19]. 

In our study, LPS treatment significantly stimulated NO, TNFα and IL-6 production in astrocytic-microglial cocultures. The LPS-stimulated production of NO, TNFα and IL-6 was much lower in isolated astrocytes. Our data is consistent with findings that quantitatively inferior microglial cells constituting the major part of pro-inflammatory micromilieu in CNS lesions [72,73,74].

LPS-induced IL-6 production in astrocytic-microglial cocultures was about two-fold higher than in isolated astrocytes. In contrast, LPS-induced NO and TNFα production differed between the cultures by a factor of about ten. This discrepancy might be explained by a relatively increased proportion of astrocytic involvement in IL-6 production. 

Whether astrocytes are able to express inducible NO synthases (iNOS), is a controversial issue. Cell culture experiments and immunohistochemical staining suggested that astrocytes cannot produce NO by inducing iNOS after stimulation with LPS, but may amplify microglial NO production due to cell–cell interaction [73,75,76]. In our experiments, in contrast to these findings, LPS-activated astrocytes produced a small but clearly detectable amount of NO. The possibility of the low microglial contamination of astrocytic cultures has been discussed as an explanation for the inconsistent findings in the literature [75]. However, in the present study, the isolation of astrocytes was achieved by clodronate rather than by the commonly practiced shaking method, so that iNOS expression by at least a subgroup of astrocytes must be postulated. Nevertheless, our data support a predominant role of microglia in the production of NO after LPS stimulation.

The basal production of NO, TNFα and IL-6 was low to not detectable in all cultures studied, and was not affected by abn-CBD. In cocultures of astrocytes and microglia, abn-CBD concentration-dependently reduced LPS-stimulated NO and TNFα production, while IL-6 production was not altered. This effect was not affected by CB_2_ knockout.

Since abn-CBD has no relevant affinity for the classic eCB receptors, there is some uncertainty regarding its molecular target structure [42,77]. Our data supports a lack of involvement of the CB_2_ receptor in abn-CBD-mediated effects. With regard to the vascular effects of abn-CBD, an orphan G-protein coupled abn-CBD-sensitive receptor was characterized [41,42,78]. Since the neuroprotective effects of abn-CBD in the OHSC model were abrogated by the abn-CBD-sensitive receptor antagonist O-1918, it can be assumed that they are mediated by the same receptor [53]. The occurrence of the putative receptor on microglial cells has already been pharmacologically characterized due to its influence on migration [44,45]. In this context, the putative abn-CBD-sensitive receptor represents a potential common target of abn-CBD and eCBs.

Microglial iNOS induction and TNFα production are associated with neurotoxic effects in the context of CNS lesions. Excessive glial NO production may interfere with neuronal cell respiration, leading to excitotoxicity through the induction of neuronal and astrocytic glutamate release, while iNOS inhibition was neuroprotective [14,17,79,80]. TNFα intervenes in further inflammatory process and stimulates astrocytes to produce IL-6 [81]. It has been found that TNFα inhibits astrocytes in their ability to support neuronal survival and neurite outgrowth [19]. The cytokine can act pro-apoptotic on neurons and inhibit the reparative sprouting of neurites [82,83]. Accordingly, reducing the production of NO and TNFα may positively affect the survival of CNS structures after injury.

In cell culture experiments, the neurotoxicity of conditioned medium derived from LPS-activated BV2-microglia was reduced by abn-CBD [64]. The reduction in microglial NO and TNFα production measured in our experiments may explain the reduced neurotoxicity. Our recent findings may also explain the results from excitotoxically lesioned OHSC, where the neuroprotective effects of abn-CBD were dependent on the presence of microglial cells [53]. In the in vivo studies in mice, abn-CBD reduced plasma TNFα levels after systemic LPS administration. Thus, the findings of the present experiments on the anti-inflammatory effects of abn-CBD are possibly partially transferable to peripheral leukocytes [84].

While effects of abn-CBD were independent of CB_2_ function, an overall altered immune response was observed in CB_2_ knockout cultures. Basal NO, TNFα and IL-6 levels were comparably low, but LPS-induced production of NO and TNFα was significantly lower, whereas LPS-induced IL-6 production did not differ. It is well accepted that the expression of CB_2_ receptors on microglial cells depends upon their activation state [85]. The CB_2_ receptor is involved in the modulation of inflammatory processes and microglial activity in the CNS [86]. The reduction in the LPS-induced production of NO and TNFα in CB_2_ knockout cultures was unexpected, since previous work demonstrated that CB_2_ activation is associated with decreased microglial inflammation and neuroprotection in a mouse stroke model [37]. This discrepancy may hint to model- and lesion-specific differences in immunomodulatory CB_2_ function. In vitro, microglial expression of CB_2_ receptors is the subject of pathogen- or cytokine-specific regulation [87,88,89]. In a mouse model of cerebral malaria, the CB_2_ knockout was also associated with a reduced inflammatory status, highlighting the model-dependent role of CB_2_ function [90]. A possible explanation for the discrepancies might be compensatory amplified signaling pathways. In this context, it is of interest whether the pharmacological blockade of CB_2_ mimics the effects observed in knockout animals.

The scratch-wound assay is widely used as a strongly reduced model for reactive astrogliosis and astrocytic scar formation. The lesion in this model is induced by the mechanical disruption of cell–cell contacts, as well as injury of cells in the wound area. Consecutive signals reach peripheral cells due to the syncytium-like cross-linking of astrocytes via gap-junctions and the paracrine secretion of cytokines [20,81,91,92]. In addition, the mechanical coupling of the cells may be relevant. Following injury, and in a similar manner to in vivo activation, the astrocytes next to the scratch undergo characteristic changes in terms of polarization, hypertrophy, GFAP expression, migration and proliferation [93,94]. 

In comparison between isolated astrocytes and cocultures, no changes were observed at the time points investigated. Thus, in the present model, astrocyte wound closure does not appear to be a priori affected by the presence of microglial cells. In astrocytic-microglial cocultures, treatment with 10 μM abn-CBD significantly delayed the wound closure, while in isolated astrocytes no significant effect was detectable. Therefore, abn-CBD appears to influence the astrocyte response secondarily through its influence on microglial activity. In this context, the change in microglial cytokine production by abn-CBD may be an explanatory approach. This hypothesis is supported by experiments showing inhibition of outgrowth of astrocyte processes in the scratch-wound assay by blocking antibodies against TNFα [94]. 

Quantification of scratch-wound assays is often done by microscopic measurement at one defined time point [93,95,96,97]. For the present study, a new protocol was developed using the possibilities of live cell microscopy. This allows a high temporal resolution of the underlying dynamics. Overall, the available data clarifies the advantages of the protocol using live cell microscopy compared to the classical procedure. Thus, the effect of abn-CBD would have been overlooked after 18 or 30 h, and a temporal classification of the underlying dynamics would not be possible when the endpoint analyzed only.

In summary, our experiments show that abn-CBD is a modulator of glial cell activation by differentially altering the secretion of pro-inflammatory mediators. It affects the reorganization of astrocytes after mechanical lesion. This provides new explanations for the neuroprotective potential of a promising substance for pharmacological use. Thus, in contrast to other cannabinoids, there is no CB_1_-mediated psychotropic effect [77,98]. While our data confirm the CB_2_-independence of abn-CBD-mediated effects, the cultures with CB_2_ knockout showed a differentially reduced response to LPS. Further understanding of the underlying molecular mechanisms, will also contribute to a better understanding of the eCB system and neuroinflammatory cascades in secondary damage.

## 4. Materials and Methods 

All experiments involving animal material were performed in accordance with the directive 2010/63/EU of the European Parliament and the Council of the European Union (22.09.2010) and approved by local authorities of the State of Saxony-Anhalt (permission number: I11M18) protecting animals and regulating tissue collection used for scientific purposes.

### 4.1. Preparation and Generation of Astrocytic-Microglial Cocultures and Isolated Astrocytic Cultures

Astrocytic-microglial cocultures were prepared from neonatal p0–1 C57BL/6 mice and corresponding CB_2_ receptor knockout mice [99,100]. In brief, mice were decapitated and scalp and skull were opened sagittally, laterally mobilized and removed. The brains were collected and transferred into chilled Hank’s Balanced Salt Solution (HBSS, Gibco BRL Life Technologies) containing Ca^2+^ and Mg^2+^. Under stereomicroscopic observation, the meninges, olfactory bulb, cerebellum and brainstem were removed. The brains were rinsed three times briefly with HBSS without Ca^2+^ and Mg^2+^ before treatment with a solution of trypsin (4 mg/mL; Gibco BLR Life Technologies) and DNAse (0.5 mg/mL; Worthington Biochemical) in HBSS (5 min; 37 °C). After rinsing again with HBSS without Ca^2+^ and Mg^2+^, the brains were suspended using DNAse (5 min, 20 °C). The digestion was stopped by the addition of HBSS containing Ca^2+^ and Mg^2+^, and the suspension was centrifuged. The pellet was resuspended in Dulbecco’s modified Eagle’s medium (DMEM, Gibco BRL Life Technologies) and the cell suspension transferred to Poly-L-Lysine (PLL, Biochrom)-coated culture flasks. Suspension from four brains was used for one culture flask. Until further use, the cells were incubated at 37 °C and 5% CO_2_. After two days, the cell debris was removed by washing the cultures with HBSS, followed by the addition of fresh culture medium. 

For cultivation, DMEM was used, containing 4.5 g/l glucose with the addition of 10% (*v*/*v*) fetal bovine serum (FBS, Gibco BLR Life Technologies), 1% (*v*/*v*) penicillin-streptomycin (Gibco BLR Life Technologies) and 0.1% (*v*/*v*) vitamin C (Sigma-Aldrich). Before use, the medium was heated (37 °C), pH adjusted to 7.4 and then sterile filtered. A change of culture medium was performed every other day. 

The culture conditions used cause the rapid death of neurons and oligodendrocytes, resulting in mixed cultures of astrocytes and microglia. After one week the cultures developed a stable ratio of astrocytes to microglial cells of approximately 10:1. 

After reaching confluence in the culture flasks, the cells were washed for 5 min at 20 °C with phosphate buffered saline (PBS, Gibco BLR Life Technologies) without Ca^2+^ and Mg^2+^, and detached using trypsin-ethylenediaminetetraacetic acid (EDTA) solution (Biochrom) (5 min, 37 °C). The reaction was stopped by adding culture medium and the cell suspension was centrifuged. The supernatant was discarded, the cell pellet resuspended and passaged in fresh culture medium on two new PLL-coated culture flasks. 

To obtain pure astrocytic cultures, microglia cells were depleted from the coculture by adding 10 μg/mL clodronate to the culture medium. The procedure was combined three times with the scheduled changes of culture medium. Thereafter, in the course of further cultivation, medium changes took place without the addition of clodronate. Clodronate at the concentration used leads to almost complete elimination of the microglial cells without affecting the activity or proliferation of astrocytes [101,102,103,104]

### 4.2. Cytokine and Nitrite Measurement

All measurements were performed on primary astrocytic-microglial cocultures and on isolated astrocytic cultures. In addition to cultures from wildtype animals, cocultures and isolated astrocytes obtained from CB_2_ receptor knockout animals were examined. At least four independent experiments were performed per cell culture and analyzed substance. In this context cultures of different animals are considered independent.

**Figure 7:**
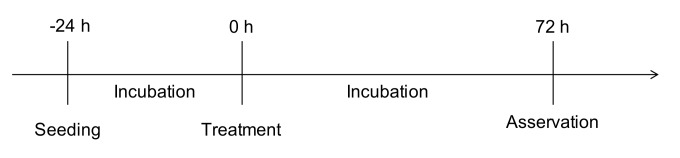
Protocol of NO and cytokine measurement

The cells were released using trypsin-EDTA solution (5 min, 37 °C) and 50,000 cells were transferred into each compartment of a 24-well plate. The number of cells was ascertained by counting the cell suspension with a Neubauer counting chamber prior to appropriate dilution. After 24 h, the culture medium was changed and the cells were treated according to the protocol (Table 1). Subsequently, the cells were incubated for 72 h with the treatment substances (37 °C, 5% CO_2_). The supernatants were collected at the end of experiments and stored at −20 °C until further evaluation (Figure 7).

For nitrite measurements, a Griess reagent optimized for use on cell culture supernatants was used (Griess reagent modified, Sigma-Aldrich) according to manufacturer’s instructions. Prior to measurement, standard concentrations were prepared as a dilution series of a 100 μM solution of sodium nitrite in culture medium at concentrations of 100 μM, 50 μM, 25 μM, 12.5 μM, 6.25 μM, 3.125 μM, 1.5625 μM and 0 μM. Subsequently, 50 μL of each sample and the standard concentrations were transferred to the compartments of a 96-well plate and treated with the same volume of Griess reagent. All measurements were done in duplicate. After 15 min, the absorbance at 540 nm was photometrically quantified by a microplate reader.

Cytokine measurements were carried out by sandwich enzyme-linked immunosorbent assay (ELISA) [105]. Commercially available ELISA kits optimized for cell culture supernatants were used (DuoSet ELISA Development System Mouse TNFα and DuoSet ELISA Development System Mouse IL-6, R & D Systems) (Wiesbaden, Germany). All additional required materials and solutions were also purchased from the manufacturer in a set (DuoSet Ancillary Reagent Kit 2, R & D Systems). When diluting the antibodies and reagents to their target concentrations and performing the assay, the manufacturer’s recommendations were followed. In brief, antibodies against murine TNFα or IL-6 were diluted in PBS to their target concentrations and the compartments of a 96-well plate were coated with the solution. During the incubation and during all following incubation steps, the plates were stored protected from light at room temperature and sealed with a self-adhesive film. The wells were washed three times with the aid of an automatic washing device. Nonspecific binding sites were blocked by incubation with a 1% solution of bovine serum albumin (BSA) in PBS. Once the samples had thawed and the standard concentrations had been prepared, another washing step was performed. Standard concentrations were generated by a dilution series of recombinant murine TNFα or IL-6 at concentrations of 2000 pg/mL (TNFα only), 1000 pg/mL, 500 pg/mL, 250 pg/mL, 125 pg/mL, 62.5 pg/mL, 31.3 pg/mL, 15.6 pg/mL and 7.8 pg/mL (IL-6 only). Samples and standard concentrations were each pipetted twice into the compartments of the antibody-coated 96-well plate and incubated. Since the preliminary experiments showed IL-6 concentrations of the samples above the measuring range, the samples were diluted 1:10 before measurement. A second biotinylated antibody against TNFα or IL-6 was diluted to its respective target concentration and the plates were incubated with this solution after a further washing step. After fixation of the target molecules, the second antibody binds the complex. After another washing step and incubation with streptavidin-horseradish peroxidase (HRP), after washing again, a mixture of H_2_O_2_ and tetramethylbenzidine was added. This solution is the substrate of HRP, and the catalyzed reaction produces a blue reaction product, and the amount of depends on the quantity of HRP bound. The reaction was stopped by the addition of H_2_SO_4_ and the absorbances at 450 nm and 540 nm were immediately measured photometrically on the microplate reader. To correct optical errors of the 96-well plate, the measured values at 540 nm were subtracted from the values at 450 nm. 

### 4.3. Scratch-Wound Assay

**Figure 8:**
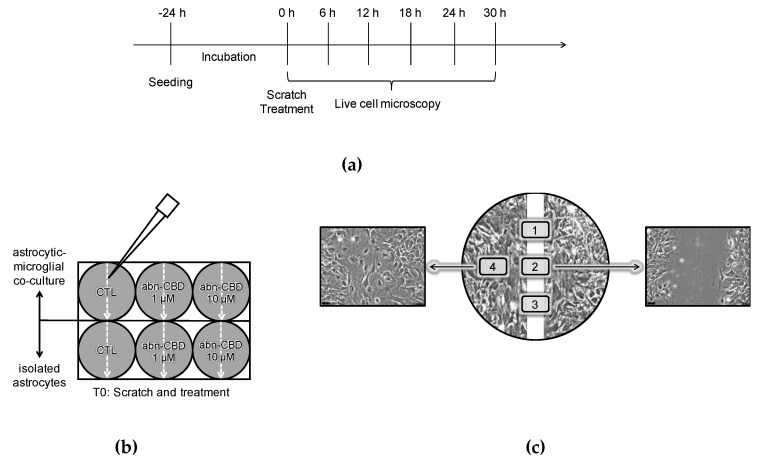
(**a**) Protocol of scratch-wound assay; (**b**) Experimental setup and treatment groups; (**c**) Selection of the observed positions

Scratch-wound assays were performed with isolated astrocytic cultures and astrocytic-microglial cocultures obtained from C57BL/6 wildtype mice. After the confluent growth in the culture flask, 500,000 cells were transferred into the compartments of a 6-well plate. The number of cells was ensured by counting the cell suspension with a Neubauer counting chamber prior to appropriate dilution. In each experiment three wells of the plate were colonized with astrocytic-microglial cocultures and the remaining three with isolated astrocytic cultures. Subsequently, the cultures were incubated for 24 h until adherence and confluence were achieved. A central, vertical and straight scratch was placed using a 10 μL pipette tip. The culture medium was replaced with fresh medium to which the respective substances were added according to the protocol. The cells were treated with abn-CBD at concentrations of 1 μM and 10 μM or methylacetate corresponding to the amount of solvent in 10 μM abn-CBD treated group (Figure 8b).

Wound closure was observed with a Leica live cell microscopy system by using a 20× phase contrast objective, while the cells were incubated constantly at 37 °C and 5% CO_2_. The examined positions were defined, so that in each compartment of the plate three overlap-free areas were imaged from the central area of the scratch. Furthermore, an additional representative Section 4 mm from the wound edge in the area of the cell monolayer was chosen to confirm the comparability of cell density (Figure 8c). The microscope software was configured to take digital pictures of the defined positions every 6 h, and the observation was continued for 30 h (Figure 8a).

A total of ten independent experiments were performed. In this context, cultures of different animals are considered independent. Positions where the wound margins were not completely visible initially or at a later time point were excluded from analysis for all time points. However, from each treatment group the images from at least eight independent experiments were evaluated. The MatLab script TScratch was used to determine the percentage image area that was not covered by cells [106]. Since automatic edge detection of the algorithm was extremely unreliable in our images, each image was manually reworked. 

### 4.4. Quantification of the Results and Statistical Analysis

For quantification of nitrite measurements mean values from duplicate measurements were formed. The standard curve was created by linear regression and the unknown values were interpolated. Since an R^2^ greater than 0.98 was achieved for each standard curve, linear regression proved to be the appropriate basis for calculations. For quantification of cytokine measurements mean values from duplicate measurements were formed. Based on the measured standard concentrations, the best possible standard curve was determined by four-parametric logistic regression, and the unknown values were interpolated. The values of IL-6 measurements were multiplied according to the 1:10 dilution. Regression of the standard curve and interpolation of the unknown values was performed with a MatLab script. The groups were tested for normal distribution by Shapiro-Wilks test. Since the existence of normal distribution was confirmed in each group, the calculated nitrite or cytokine concentrations were analyzed for differences with one-way ANOVA and subsequent Bonferroni’s post-test. For linear regression, interpolation and statistical analysis the software GraphPad Prism 5 was used.

For quantification of the scratch-wound assay the mean values were calculated from three values per well and time point. Progressive wound closure was quantified by the cumulative change of the cell-free image area compared to the cell-free image area at time point zero in the respective well. In addition, the respective changes in cell-free image area at the 6-h intervals 0 h–6 h, 6 h–12 h, 12 h–18 h and 18 h–24 h, were analyzed to characterize the temporal dynamics of wound healing and treatment effects. The groups were tested for normal distribution by the Shapiro-Wilks test. Since the existence of normal distribution was confirmed in each group, statistical analysis was performed with one-way ANOVA and Bonferroni’s post-test using GraphPad Prism 5.

### 4.5. Fluorescence Immunocytochemistry and Confocal Laser Scanning Micoscropy

Exploratory immunohistochemistry was performed on wildtype astrocytic-microglial cocultures and isolated astrocytes. Treatment of the cells and seeding on coverslips proceeded exactly as for cytokine and nitrite measurement (Section 4.2.). Cells from treatment groups 1, 3, 4 and 6 (Table 1) were analyzed. Cultures were fixed with 4% paraformaldehyde in 0.1 M phosphate buffer 72 h after treatment. After washing with PBS, the cells were incubated with normal horse serum (NHS) diluted 1:20 in PBS containing 0.03% Triton (PBS-Triton) (30 min, 20 °C). Sections were then incubated overnight with a primary mouse anti-GFAP antibody (BD Pharmingen, diluted 1:200 in PBS-Triton with 5% BSA, 20 °C). After washing with PBS-Triton (3 times for 10 min, 20 °C), incubation with secondary Alexa 568 goat-anti-mouse antibody (Invitrogen, diluted 1:200 in PBS-Triton) and FITC-conjugated *Griffonia simplicifolia* isolectin B_4_ (FITC-IB_4_, Biozol, diluted 1:50 in PBS-Triton) was performed (1 h, 20 °C). The cells were washed again with PBS-Triton (3 times for 10 min, 20 °C) and then incubated with 4′,6-diamino-2-ohenylindole (DAPI, Sigma-Aldrich, diluted 1:10.000 in aqua destillata). Sections were washed with aqua destillata (5 min, 20 °C), mounted with DAKO fluorescent mounting medium (Agilent) and analyzed by confocal laser scanning microscopy (Leica). Cellular nuclei, astrocytes and microglial cells were visualized using monochromatic light, emission filters with the specified wavelengths and 40× objective. 

## Figures and Tables

**Table 1:** Treatment groups for cytokine and nitrite measurement

Group	Substances
1	CTL 0.66 µL/mL methylacetate (MA)
2	Abn-CBD 1 µM + 0.6 µL/mL MA
3	Abn-CBD 10 µM including 0.66 µL/mL MA
4	LPS 10 ng/mL + 0.66 µL/mL MA
5	LPS 10 ng/mL + abn-CBD 1 µM + 0.6 µL/mL MA
6	LPS 10 ng/mL + abn-CBD 10 µM including 0.66 µL/mL MA

All groups received the same concentration (0.66 µL/mL) of the solvent methylacetate (MA) as required for 10 µM abnormal cannabidiol (abn-CBD) groups.

## Supplementary Materials

The following are available online at https://www.mdpi.com/1420-3049/25/3/496/s1, Figure S1: Representative images from exemplary immunohistochemistry; Video S2: Example video of scratch-wound assay.
