# Supplementary material for: Abnormal Cannabidiol Affects Production of Pro-Inflammatory Mediators and Astrocyte Wound Closure in Primary Astrocytic-Microglial Cocultures
Source: Molecules. 2020 Jan 23;25(3):496. doi: 10.3390/molecules25030496 (PMC7037200; doi:10.3390/molecules25030496)
Supplement: Supplementary file 1 [file molecules-25-00496-s001.zip › supplementary/Suppl fig 1_664770_molecules_Corrected Proof.pdf]

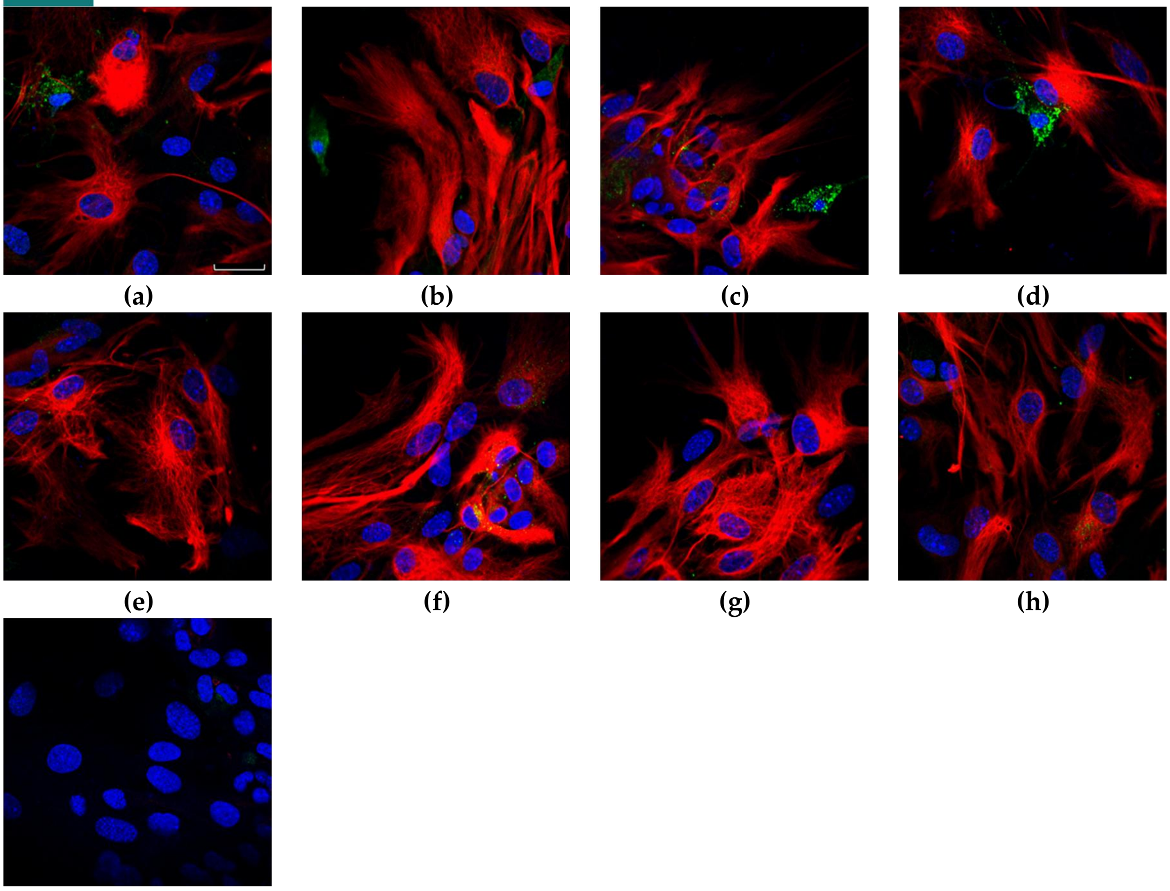

Figure S1. Representative images from exemplary immunohistochemistry. Exemplary labeling of GFAP, IB_4 and nuclei in astrocytic-microglial co-cultures **(a-d)** and isolated astrocytes **(e-h)**. Red fluorescence points to GFAP-positive astrocytes, green fluorescence to IB_4-positive microglia, while DAPI-labeled nuclei are blue. The treatment groups CTL **(a, e)**, 10 µM Abn-CBD **(b, f)**, 10 ng/ml LPS **(c, g)** and 10 µM Abn-CBD + 10 ng/ml LPS were examined. Negative control (without primary antibody) confirming the specificity of the secondary antibody **(i)**. (Scalebar in **(a)** =25 µm, all images same scale)
